# Low Dose Colonization of Broiler Chickens With ESBL-/AmpC- Producing *Escherichia coli* in a Seeder-Bird Model Independent of Antimicrobial Selection Pressure

**DOI:** 10.3389/fmicb.2019.02124

**Published:** 2019-09-13

**Authors:** Caroline Robé, Anja Blasse, Roswitha Merle, Anika Friese, Uwe Roesler, Sebastian Guenther

**Affiliations:** ^1^Institute for Animal Hygiene and Environmental Health, Freie Universität Berlin, Berlin, Germany; ^2^Institute for Veterinary Epidemiology and Biostatistics, Freie Universität Berlin, Berlin, Germany; ^3^Institute of Pharmacy, Pharmaceutical Biology, Universität Greifswald, Greifswald, Germany

**Keywords:** ESBL, AmpC, *Escherichia coli*, broiler chicken, colonization, antimicrobial selection pressure, seeder bird model

## Abstract

Extended-spectrum beta-lactamase- (ESBL-) and AmpC beta-lactamase- (AmpC-) producing Enterobacteriaceae pose a risk for both human and animal health. For livestock, highest prevalences have been reported in broiler chickens, which are therefore considered as a reservoir of multidrug-resistant bacteria. The possibility of transfer to humans either by a close contact to colonized broiler flocks or through contaminated retail meat results in the necessity to develop intervention measures for the entire broiler production chain. In this regard, a basic understanding of the colonization process is mandatory including the determination of the minimal bacterial load leading to a persistent colonization of broiler chickens. Therefore, we conducted a bivalent broiler colonization study close to real farming conditions without applying any antimicrobial selection pressure. ESBL- and AmpC- negative broiler chickens (Ross 308) were co- colonized on their third day of life with two strains: one CTX-M-15-producing *Escherichia coli*-ST410 and one CMY-2/*mcr*-1-positive *E. coli*-ST10. Colonization was assessed by cloacal swabs over the period of the trial, starting 24 h post inoculation. During the final necropsy, the contents of crop, jejunum, cecum, and colon were quantified for the occurrence of both bacterial strains. To define the minimal oral colonization dosage 10^4^ to 10^1^ colony forming units (cfu) were orally inoculated to four separately housed broiler groups (each *n* = 19, all animals inoculated) and a dosage of already 10^1^ cfu *E. coli* led to a persistent colonization of all animals of the group after 3 days. To assure stable colonization, however, a dosage of 10^2^ cfu *E. coli* was chosen for the subsequent seeder-bird trial. In the seeder-bird trial one fifth of the animals (seeder, *n* = 4) were orally inoculated and kept together with the non-inoculated animals (sentinel, *n* = 16) to mimic the route of natural infection. After 35 days of trial, all animals were colonized with both *E. coli* strains. Given the low colonization dosage and the low seeder/sentinel ratio, the rapid spread of ESBL- and AmpC- producing Enterobacteriaceae in conventional broiler farms currently seems inevitably resulting in an urgent need for the development of intervention strategies to reduce colonization of broilers during production.

## Introduction

Antimicrobial resistance is an increasing problem in veterinary and human medicine and the spread of extended-spectrum beta-lactamase- (ESBL-) and AmpC beta-lactamase- (AmpC-) producing Enterobacteriaceae was demonstrated in wildlife (Guenther et al., 2011; Alcalá et al., 2016), livestock (Laube et al., 2013; Liebana et al., 2013; Hille et al., 2018), and companion animals (Rubin and Pitout, 2014; Schaufler et al., 2015; Kaspar et al., 2019) as well as in retail meat (Cohen Stuart et al., 2012; Kola et al., 2012) and out of human origin (Pitout, 2013; Valenza et al., 2014; Mazzariol et al., 2017).

Livestock animals, including broiler chickens, show high prevalences (EFSA, 2019) and are considered to be a reservoir of ESBL- and AmpC- producing enterobacteria (Costa et al., 2009; Reich et al., 2013). Recently, it was shown by Projahn et al. (2017) and Daehre et al. (2018b) that transmission of resistant bacteria can take place at a very early stage of the broiler production chain: pseudo-vertical from the parent flock to the eggs in the hatchery and horizontal through the contaminated farm environment 24 h after the placing of the broiler chickens. Genes encoding ESBLs and AmpCs are frequently located on mobile genetic elements such as plasmids (Carattoli, 2013). Commonly detected resistance genes include *bla*_CTX−M−1_, *bla*_CTX−M−14_, *bla*_CTX−M−15_, *bla*_SHV−12_, and *bla*_CMY−2_ in livestock and companion animals (Ewers et al., 2012). The question of a possible transfer of resistance from animals to humans remains controversial and constitutes a major concern of public health (Cortés et al., 2010; Leverstein-van Hall et al., 2011; Ewers et al., 2012; Dorado-García et al., 2018; Pietsch et al., 2018). Close contact of humans to colonized animals (Dierikx C. et al., 2013; Huijbers et al., 2014) and the consumption of contaminated meat (Vincent et al., 2010; Leverstein-van Hall et al., 2011) are considered to be especially important risk factors, and have been thoroughly investigated.

By contrast, to our knowledge, there have not been any studies investigating the minimal bacterial load that is necessary for a stable colonization of broiler chickens (Daehre et al., 2018b). Therefore, the aim of our study was to define the minimal colonization dosage of broiler chickens with ESBL- and AmpC- producing *Escherichia coli (E. coli)* without applying any antimicrobial selection pressure. In the second step, a seeder- bird colonization model that mimics real farming conditions was conducted for the investigation of the spread of ESBL- and AmpC- producing *E. coli* in flocks. These investigations paved the way for the follow-up studies that are currently being conducted at our institute and aim to examine potential intervention strategies regarding hygiene- and management measures to reduce the colonization of broiler chickens with ESBL- and AmpC- producing Enterobacteriaceae.

## Materials and Methods

### Ethics Statement

This study was carried out in accordance with the National Animal Protection Guidelines. The protocol was approved by the German Animal Ethics Committee for the protection of animals of the Regional Office for Health and Social Affairs Berlin (“Landesamt für Gesundheit und Soziales,” LAGeSo, registration number G 0193/16).

### Housing Conditions

All trials were conducted at the experimental facilities of the Center for Infection Medicine of the Freie Universität Berlin, department of Veterinary Medicine. The animals were kept in controlled rooms with attached separate lockrooms for changing of clothes and shoes. Before starting the trial, rooms were cleaned and disinfected with hydrogen peroxide fumigation and the complete experimental setup was tested for the absence of ESBL-/AmpC- producing bacteria (see “ESBL-/AmpC- status prior to the trial”). An individual ventilation was achieved by using HEPA filter. The study was structured in two parts: a colonization dosage part followed by a seeder-bird colonization model.

For each trial, eggs of the breed Ross 308, received from a commercial hatchery in Germany, were hatched in-house for 21 days. The first disinfection of the eggs using formaldehyde gas was performed at the hatchery and the second disinfection with WESSOCLEAN® K 50 Gold Line containing 2.37% hydrogen peroxide and 0.015% peracetic acid (Wesso AG, Hersbruck, Germany) following the transportation to the experimental facilities before incubation in a separate hatcher. After hatch, broilers were conventionally housed in with a stocking density of 39 kg/m^2^, fresh litter once at the beginning of the trial, no enrichment and conventional feed and water *ad libitum*. The feeding regime included a starter feed and a grower feed with coccidiostats (decoquinate and narasin/nicarbazin). The finisher feed did not include any coccidiostats and was fed 5 days prior to necropsies. The feed did not contain any antimicrobial agents.

First, four colonization dosage trials were performed. Each of the four groups consisted of 19 chickens. Every group was kept separately in an experimental room for a period of 2 weeks in aviaries to define the minimal oral colonization dosage. Following these colonization dosage experiments, 20 chickens were kept in floor keeping for the seeder-bird model trial. For the seeder- bird trial, four chickens were orally inoculated (seeder birds) with the minimal oral colonization dosage determined previously and kept together with 16 non-inoculated animals (sentinel birds). During the seeder-bird trial, the chickens were kept up to a weight of two kilograms, corresponding to the duration of 5 weeks.

In the later course of the colonization dosage trial using 10^2^ cfu/*E. coli* (day 11) and the seeder-bird trial (day 16) each time one animal died without any sign of infection or illness.

### Bacterial Colonization Strains

Two avian *E. coli* strains were used for co-colonization of the birds. Both strains were originally isolated from healthy chickens during a previous research project on ESBL- producing *E. coli* in chickens in 2011 (Falgenhauer et al., 2016; Hering et al., 2016). Both strains were investigated using whole-genome analysis as described in Falgenhauer et al. (2016). One strain is an ESBL- producing *E. coli* {CTX-M-15 [chromosomally encoded (Arredondo-Alonso et al., 2018)]; multilocus sequence type ST410; phylogenetic group B1; internal number 10716; published as R56 in Falgenhauer et al. (2016)} with resistances to cephalosporins and enrofloxacin, and the other strain is an AmpC- producing *E. coli* {CMY-2 [plasmid encoded (Arredondo-Alonso et al., 2018)]; ST10; phylogenetic group A; internal number 10717}, resistant to cephalosporins and colistin, as shown in Table 1. In addition, phenotypic antimicrobial resistance analysis was performed by using VITEK 2 system (bioMérieux, Marcy-l'Étoile, France). These avian commensal *E. coli* strains were selected because of their resistance profile, representative for the situation in German chicken production and their ability to colonize broiler chickens digestive tract.

**Table 1:** Characteristics of *E. coli* strains used for colonization dosage trials (10^1^−10^4^ cfu *E. coli*) and seeder-bird colonization model.

**Strain**	**Origin**	**MLST**	**Phylogenetic group**	**ESBL-/AmpC type**	**None ESBL-/AmpC resistance genes**	**Phenotypic resistances**
10716	Chicken	ST410	B1	${\mathrm{bla}}_{\text{CTX}-\text{M}-15}^{*}$	aadA1^*^, *aac*(3)-IIa^*^, *aadA5*^+^, *aadB*^+^, *mph*(A)^+^, catA1^*^, *floR*^+^, *sul1*^+^, *tet*(A)^+^, *dfrA17*^+^	ATM, CAZ, CIP, CTX, GM, PIP, TZP, SXT, TM
10717	Chicken	ST10	A	${\mathrm{bla}}_{\text{CMY}-2}^{+}$	${\mathrm{bla}}_{\text{TEM}-1}^{+}$, *aadA1*^+^, *aadA2*^+^, *mcr-1*^+^, *cmIA1*^+^, *sul3*^+^	ATM, CAZ, CTX, CST, PIP, TZP

*10716 = ESBL- E. coli R56, 10717 = AmpC- E. coli G148-1 (Falgenhauer et al., 2016; Hering et al., 2016)*;

* *chromosomally encoded*;

*^+^plasmid encoded [determination using mlplasmids (Arredondo-Alonso et al., 2018)]; ATM, aztreonam; CAZ, ceftazidim; CIP, ciprofloxacin; CTX, cefotaxime; CST, colistin; GM, gentamicin; PIP, piperacillin; TZP, piperacillin/tazobactam; TM, tobramycin; SXT, trimethoprim/sulfamethoxazole*.

Microorganisms were stored at −80°C in Luria Bertani broth (LB; Carl Roth, Karlsruhe, Germany) containing 20% glycerol (Carl Roth, Karlsruhe, Germany). For the preparation of the bacterial suspension both bacterial strains were streaked out on columbia agar containing 5% sheep blood (Oxoid, Wesel, Germany) and were incubated for 24 h at 37°C. Overnight cultures originating from a single colony from each strain were grown in 5 ml LB broth. On the following colonization day, 40–60 μl of these bacterial suspensions were seeded in 5 ml of fresh LB broth each until the desired optical density of 0.04 (OD_600_) was obtained. The bacterial cultures were grown at 37°C and 200 rounds per minute (rpm) to the optical density of 1.0 (OD_600_), corresponding to 1 × 10^8^ colony forming units (cfu) per ml. One ml per culture was centrifuged at 7,000 rpm for 10 min at 4°C, the supernatant was removed and the cells were resuspended in 1 ml phosphate buffered saline (PBS; Phosphate Buffered Saline tablets, Oxoid, Wesel, Germany). The desired colonization dose was adjusted via a 10-fold dilution series in PBS. For co-colonization, both dilutions were mixed, placed on ice, and used for inoculation of broilers within 30 minutes. The concentration of the colonization dose was verified by direct plating of appropriate dilutions of the inoculum.

### Oral Colonization of Broilers

On the third day of life, broilers were orally inoculated into the crop with 200 μl of bacterial suspension in PBS containing a mixture of both bacterial strains in equal parts. For the colonization dosage trials, all animals of the group were orally inoculated, starting with a colonization dosage of 10^4^ cfu *E. coli*/200 μl followed by 10^3^, 10^2^, and 10^1^ cfu *E. coli*/200 μl in the following experiments to verify the cfu that is necessary to colonize all animals within 24 h *post inoculation* (*p.i*.).

In contrast to the colonization dosage trials, not all animals of the seeder-bird model trial were orally inoculated. The inoculation was performed at the ratio of 1:5 (seeder: sentinel), resulting in four seeders, which are expected to colonize the 16 non-inoculated, susceptible broilers (sentinels) over the period of the trial. From the beginning of the trial, the seeder- and sentinel birds were housed in together. Similarly to the dosage trials, all inoculated animals should be colonized within 24 h *p.i*.

### Samplings and Analyses

Samples were initially processed on chromogenic agar (CHROMagar Orientation, Mast Diagnostica, Reinfeld, Germany) for a rapid identification of *E. coli* colonies. Confirmation of single bacterial *E. coli* colonies with a typical morphology and of all untypical colony morphologies was performed using MALDI- TOF (MALDI Microflex LT® and Biotyper database® Bruker Daltonics, Bremen, Germany). Phenotypic antimicrobial resistance analysis of randomly picked colonies of the cecum samples obtained in necropsy was performed by using VITEK 2 system to confirm their identity as the inoculated ESBL- and AmpC- strains.

#### ESBL-/AmpC- Status Prior to the Trial

Before starting a trial, the complete experimental setup was tested for the absence of ESBL-/AmpC- producing bacteria after the introduction of the litter, feed, and water into the experimental room. Walls/doors, floor, table, heating lamps, feeding-, and drinking troughs were sampled with sterile and moistened gauze swabs. For moistening, 5 ml PBS were used. In addition, 5 g of litter, and feed were collected. Following the sampling, the gauze swabs, litter, and feed were transferred into 50 ml LB broth in each case and incubated for 24 h at 37°C before 10 μl were streaked out on chromogenic agar containing 2 μg/ml cefotaxime (AppliChem, Darmstadt, Germany) and incubated for 24 h at 37°C.

Directly after hatching, the absence of ESBL and AmpC- producing bacteria in the egg shells was confirmed. Five egg shells were crushed, transferred into 50 ml LB broth and incubated for 24 h at 37°C before 10 μl were streaked out on chromogenic agar containing 2 μg/ml cefotaxime and incubated for 24 h at 37°C.

The absence of ESBL and AmpC in the 1 day-old broiler chickens was confirmed by cloacal swabs of all individually tagged animals. Swabs were transferred into reaction tubes containing 500 μl PBS and were thoroughly vortexed. Fifty microliter were streaked out onto chromogenic agar containing 2 μg/ml cefotaxime and incubated for 24 h at 37°C.

#### Sampling During the Trial

Colonization of broilers was analyzed via cloacal swabs during the trial starting 24 h and 72 h *p.i*. and followed by samplings of each individual three times in the second week of a trial, followed by two samplings a week up to the end of a trial. Swabs were transferred into reaction tubes containing 500 μl PBS and were thoroughly vortexed. Fifty microliter were streaked out onto agar and incubated for 24 h at 37°C for semiquantitative analysis. A subjective measurement using eight categories from zero (“no growth”) to seven (“overgrown”) was applied for the evaluation of the colonization (Figure S1) and a set of four chromogenic agar plates was used. One plate without selective media for the total count of *E. coli* colonies (positive control). One plate containing 2 μg/ml cefotaxime and 4 μg/ml enrofloxacin (Sigma- Aldrich, Steinheim, Germany) for the growth of the ESBL- *E. coli* 10716 and another plate containing 2 μg/ml cefotaxime and 7 μg/ml colistin (Carl Roth, Karlsruhe, Germany) for the growth of the AmpC- *E. coli* 10717. A fourth plate contained all three antibiotics in the given concentrations (negative control).

#### Finalization of the Trial

Following each trial, a necropsy of all broilers was performed on day 14 in the colonization dosage trials and on day 35 in the seeder-bird model trial. Contents of crop, jejunum, cecum, colon as well as organ samples of liver and spleen were analyzed using the plate set described above.

The samples of crop, jejunum, cecum, and colon were quantified. First, up to one gram of the content was weighed out in a reaction tube and PBS was added at the ratio of 1:2, followed by a dilution series in PBS, depending on the expected bacterial growth. Finally, 100 μl each of the suspension were plated on the plate set, as described above. For quantification, a minimum of two different dilution levels were counted.

The organ samples of liver and spleen were qualitatively analyzed via direct processing. For this, the organs were cut in half with sterile scissors, pressed on the agar and spread out with an inoculation loop (Selbitz et al., 2015). All samples were incubated for 24 h at 37°C.

### Statistical Analysis

Sample size calculation was based on the hypothesis that the logarithmic mean values in the colonized ceca were equal in each of the different colonization dosage groups (NCSS PASS 14.0). Ceca were selected because ceca are known to be the reservoir for ESBL- *E. coli*. Equivalence was defined as follows: the 95% confidence interval (CI) of the investigated group is within one log unit cfu/g cecal content compared to the mean value of the minimal colonization dosage group. Standard deviation of log 0.9 cfu/g cecal content was assumed. To ensure alpha error of 0.05 and power of 0.90, 19 animals per group were required. Twenty animals were used for the seeder-bird colonization model to have a comparable group size for the defined inoculation ratio of 1:5 (4 seeder birds: 16 sentinel birds).

Statistical analysis was performed by using Excel 2013 (Microsoft Corporation, Washington, USA) and SPSS Statistics 25 (IBM, New York, USA). To obtain normally distributed data, all bacteriological results were log10 transformed. CI of proportions were calculated using Clopper-Pearson method.

To compare the level of colonization in between the four colonization dosage groups, equivalence testing (Thrusfield, 2007) was performed. For this test, a margin of one log unit difference between the groups was defined acceptable to be equal. It was tested whether the margins of the 95% CI are within the range of one log unit.

Differences between different groups regarding the logarithmic bacteriological mean values of the samples were investigated using *t*-test for independent samples: first, the colonization levels between the colonization dosage group 10^2^ cfu/*E. coli* and the seeder-bird colonization model were investigated separately for the locations crop, jejunum, cecum, and colon. Second, the results of the seeder- and sentinel birds of the seeder-bird model were compared. The probability level of 0.05 was used to denote significance.

## Results

### Colonization Dosage

By applying a series of colonization dosages ranging from 10^4^, 10^3^, and 10^2^ to 10^1^ cfu *E. coli*, we were able to demonstrate that a dosage of 10^2^ cfu *E. coli* per animal led to an almost complete colonization of a broiler group within 24 h *p.i* (Figure S1). Even a dosage of 10^1^ cfu *E. coli* was sufficient to colonize the majority of the animals within 24 h *p.i*. For the colonization dosage trials, all animals in a group were orally inoculated with both bacterial strains on their third day of life and the successful colonization was initially assessed via cloacal swabs 24 h *p.i*. For colonization dosages 10^4^ and 10^3^ cfu *E. coli*, all animals were colonized within 24 h *p.i*. up to the end of the trial. Continuing with a dosage of 10^2^ cfu *E. coli*, only one animal was tested negative 24 h *p.i*. for one of the strains (AmpC- *E. coli* 10717, 95%CI: 73.97–99.87%). At the second sampling 72 h *p.i*., however, all animals inoculated with 10^2^ cfu *E. coli* were colonized over the entire period of the trial. The dosage trials were completed with 10^1^ cfu *E. coli* resulting in a colonization rate of 84% (ESBL- *E. coli* 10716, 95%CI: 60.42–96.62%) and 95% (AmpC- *E. coli* 10717, 95%CI: 73.97–99.87%) of the animals positive for the strains 24 h *p.i*. In this group, complete colonization was detectable within 72 h *p.i*. up to the end of the trial. Based on the results obtained from cloacal swabs during the trial, a minimal dosage of 10^2^ cfu *E. coli* was necessary to colonize a broiler group in the given experimental setup within 24 h *p.i*.

**Figure 1:**
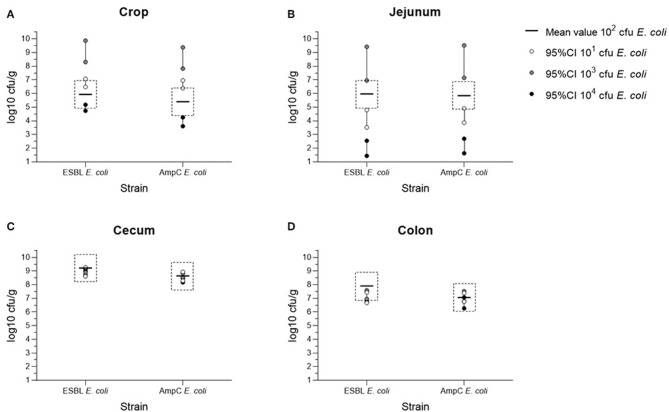
Equivalence testing of ESBL*- E. coli* 10716 and AmpC- *E. coli* 10717 in samples of crop **(A)**, jejunum **(B)**, cecum **(C)**, and colon **(D)** in between the four colonization dosage groups (10^1^-10^4^ cfu/*E. coli*) at necropsy. 95%CI = 95% confidence interval; all data shown are log10 transformed (log10 cfu/g); margin of <1 log of 95%CI of dosage groups 10^1^, 10^3^, and 10^4^ cfu/*E. coli* to the mean value of the minimal colonization dosage group (10^2^ cfu/*E. coli*) shows equivalence.

**Figure 2:**
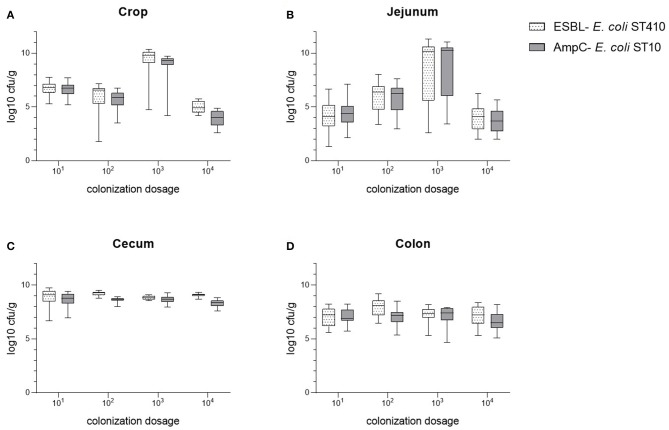
Comparison of the bacterial counts of ESBL- *E. coli* 10716 and AmpC- *E. coli* 10717 in samples of crop **(A)**, jejunum **(B)**, cecum **(C)**, and colon **(D)** in between the four colonization dosage groups (10^1^-10^4^ cfu/*E. coli*) at necropsy.

The bacterial counts of the cecum samples obtained at the necropsies of all four trials (10^4^ to 10^1^ cfu *E. coli*) were of equal value in the equivalence test. In contrast to this, samples of crop, jejunum, and colon were non- equivalent (Figure 1). The mean colonization values of the digestive tract samples for both bacterial strains are summarized in Figure 2 and Table S1. Overall, maximum values were attained in cecum samples with mean values between 8.84 to 9.22 log10 cfu ESBL- *E. coli*/g cecal content and 8.32 to 8.69 log10 cfu AmpC- *E. coli*/g cecal content, except for the content of crop and jejunum in the dosage trial using 10^3^ cfu *E. coli*. Compared to the cecum samples, lower mean values occurred in colon samples and lowest mean values were evident in the content of crop and jejunum (Table S1). Independent from the original colonization dosage, the results of cecum samples were equivalent with a deviation less than one log unit to the values of the colonization dosage of 10^2^ cfu *E. coli* and every broiler was colonized with both bacterial strains 2 weeks *p.i*. For samples of crop, jejunum and colon, equivalence between dosages was not shown. The values of crop and jejunum samples differed in each trial. Highest values were given in the dosage trial using 10^3^ cfu *E. coli*, lowest values occurred while using 10^4^ cfu *E. coli*. In contrast to this, less variation was given in colon samples with mean values between 7.05 to 7.91 log10 cfu ESBL- *E. coli*/g colon content and 6.67 to 7.15 log10 cfu AmpC- *E. coli*/g colon content, but without showing equivalence.

In conclusion, the colonization of the cecum with both bacterial strains 2 weeks after inoculation with 10^2^ cfu *E. coli* was equal to the colonization after inoculation with 10^4^, 10^3^, and 10^1^ cfu *E. coli*. In contrast to these correlating results of cecum samples obtained at necropsy, we observed lower percentages of colonized animals 24 h after inoculation by using 10^1^ cfu *E. coli* compared to the other tested dosages. Based on these results, a dosage of 10^2^ cfu *E.coli* was chosen for the following seeder- bird colonization model trial balancing between a low dose, comparable to the situation in the course of natural infection and a colonization of the animals during the trial.

### Seeder-Bird Colonization Model

A ratio of 1:5 orally inoculated (seeder) to non- inoculated broilers (sentinel) and a dosage of 10^2^ cfu ESBL-/AmpC- *E. coli* were sufficient to colonize an entire broiler group within 72 h (Figure S1). The seeder birds were completely colonized 24 h *p.i*. with both bacterial strains. At this early time point 69% of the 16 sentinels were tested positive for the ESBL- *E. coli* strain 10716 (95%CI: 41.34–88.98%) and 50% positive for AmpC- *E. coli* 10717 (95%CI: 24.65–75.35%). Three days *p.i*., at the second sampling, all animals of the broiler group were colonized with both bacterial strains up to the end of the trial. As a result, an oral colonization of one fifth of a broiler group with 10^2^ cfu *E. coli* on their third day of life resulted in colonization of the entire broiler group.

At necropsy, cecum colonization of all seeder- and sentinel birds was observed, with mean values of 6.69 log10 cfu ESBL- *E. coli*/g cecal content and 6.57 log10 cfu AmpC- *E. coli*/g cecal content (Table S1). Furthermore, no significant difference in bacterial counts between seeder birds and sentinel birds was evident at the end of the trial (*t*-test *p*-values > 0.05). Corresponding to the colonization dosage trials, the maximum values of *E. coli* were found in the cecum irrespective of being a seeder- or sentinel bird. Compared to the cecum samples, reduced values occurred in colon samples and lowest mean values were evident in crop and jejunum samples. Similarly, no association with the origin of the bird as a seeder- or sentinel was observed (t-test *p*-values > 0.05).

**Figure 3:**
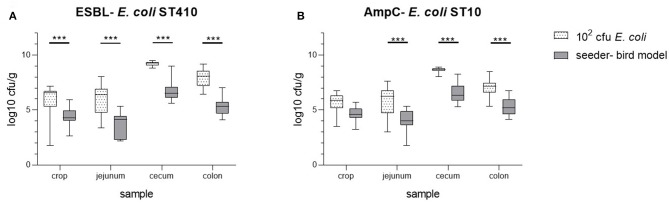
Comparison of the bacterial counts of ESBL- *E. coli* 10716 **(A)** and AmpC- *E. coli* 10717 **(B)** in samples of crop, jejunum, cecum, and colon at necropsy between the minimal colonization dosage group (10^2^ cfu/*E. coli*; necropsy on day 14; all animals inoculated on third day of life) and the seeder-bird model (necropsy on day 35; inoculation ratio of 1:5 on third day of life). ***p < 0.001 (*t*-test).

However, compared to the dosage trial using 10^2^ cfu *E. coli*, the colonization in different parts of the digestive tract of the seeder-bird group was significantly lower (*t*-test *p*-values <0.001; Figure 3). In all necropsy samples taken, we observed lower total numbers of both bacterial strains 5 weeks *p.i*. in the seeder-bird model compared to the initial colonization dosage trial with a duration of 2 weeks. In summary, a comparable colonization of all seeder- and sentinel birds in a group was achieved by an oral colonization dosage of 10^2^ cfu *E. coli* in combination with an oral inoculation of one fifth of the broiler chickens on their third day of life.

To conclude, without any antimicrobial selection pressure by antibiotic usage, both commensal ESBL- and AmpC- producing *E. coli* strains colonized all broilers digestive tract independent of being an orally inoculated seeder bird or a susceptible sentinel bird. For all four colonization dosage trials and the seeder-bird trial we did not detect any colonization of liver or spleen with our bacterial strains tested. By analyzing the respective antimicrobial resistance patterns via VITEK 2 of the strains derived from the cecum samples at the necropsies, we did not observe major changes in the resistance profile of the inoculation isolates to the isolates at the end of the trial. The rate of transfer of resistance plasmids to other strains can therefore be considered to be of low importance, as some of our resistance markers were also chromosomally encoded, such as fluoroquinolone resistance. In addition, we did not observe changes in the morphotypes of the isolates during the trials.

## Discussion

Our study points out that (i) a colonization dosage of 10^2^ cfu *E. coli* per animal is sufficient for a successful long-time colonization of broiler chickens and (ii) a ratio of 1:5 inoculated seeder birds to non- inoculated sentinel birds is sufficient to colonize a complete broiler group even in the absence of antimicrobial selection pressure. In addition, we also demonstrated that even a very low colonization dosage of 10^1^ cfu *E. coli* leads to a colonization of broilers in the given experimental setup using a study design based on parameters of real farming conditions. The animals were treated equally to those in conventional fattening farms. This includes a stocking density of 39 kg/m^2^, fresh litter once at the beginning of the trial, no enrichment and conventional feed and water *ad libitum*. Neither the utilized eggs and animals, nor the feed, water or litter were additionally processed for the reduction of germs than usual in the broiler production. In agreement, we received all eggs, litter and feed from a commercial poultry producer in Germany. To ensure comparable conditions in all trials, we used batches from one single producer and all trials were conducted within 6 months. Hence, an interaction between the animals and their surrounding environment including the present bacterial spectrum was possible before inoculation of the broiler chickens with the bacterial strains (Apajalahti et al., 2004; Ballou et al., 2016; Kumar et al., 2018). As the surrounding can be a source of ESBL- and AmpC- acquisition (Dierikx C. M. et al., 2013; Daehre et al., 2018b), we tested the complete experimental setup for the absence of ESBL-/AmpC- producing bacteria prior to the trial to exclude the possibility of a colonization out of the environment.

We inoculated our broiler chickens on the third day of life with two bacterial strains, harboring frequently detected ESBL- and AmpC- resistance profiles in fattening chickens (Ewers et al., 2012; Valentin et al., 2014). This is in accordance with numerous studies assuming a very early colonization of the broilers in the production chain. Projahn et al. (2017) describes a possible transfer of resistant bacteria already in the hatchery and during the transportation of the hatchlings to the fattening farm (Projahn et al., 2018). Furthermore, the fattening stable itself (Daehre et al., 2018b) or the surrounding areas (Laube et al., 2014) are potential sources of the acquisition of resistant bacteria. At the same time, investigations of the ESBL- and AmpC- prevalence of day- old broiler chickens are heterogeneous, varying between 95 and 0% in the analyzed broiler flocks (Laube et al., 2013; Daehre et al., 2018b). Thereby, both studies analyzed seven broiler flocks via cloacal swabs of 20 (Laube et al., 2013) or 40 broiler chickens (Daehre et al., 2018b) and both used a pre-enrichment in LB to determine the ESBL- and AmpC- prevalence. The wide variations may be caused by the selected sampling times. Laube et al. (2013) sampled within the first two days of life. In contrast to this, Daehre et al. (2018b) sampled immediately after placing the broiler chickens into the stable. However, it must be noted, that the overall prevalence of the investigated flocks is higher in the study by Laube et al. (2013). As it is known, compared to other species, the relation between the body length and the length of the gastrointestinal tract of chickens is relatively short with a rapid passage time (Hughes, 2008). To attain a reliable detection of our bacterial strains, we decided for an initial validation of our bacterial strains 24 h after inoculation. At this early time point, more than half of the sentinel birds in the seeder-bird trial had been tested positive for the ESBL- and AmpC- producing strains. This indicates a double passage of the gastrointestinal tract of firstly the seeder birds and following the sentinel birds within 24 h. These results were obtained by direct processing of the cloacal swabs without enrichment. To ensure a strong colonization of the broiler chickens, a direct detection of the bacterial strains is required. At the same time, a swab sampling provides only limited information about the intestinal colonization of the birds. The detection is strongly influenced by the amount of feces on the swab. Thus, our detection limit is relatively high, depending on the cloacal excretion during the sampling. For this reason, we decided for a minimal colonization dosage of 10^2^ cfu *E. coli* even if one broiler chicken was tested negative for one of the bacterial strains 24 h *p.i*.

As this study aims to mimic the route of natural infection with ESBL- and AmpC- producing Enterobacteriaceae, a housing of all broiler chickens immediately from the beginning of the trial in one pen is important. Under real farming conditions, it is not possible to distinguish between colonized and non-colonized birds as well. Consequently, a distinction between colonization with the bacterial strains via inoculation or through the oral uptake out of the contaminated environment was not aimed in our trial. Although we cannot exclude that the colonization via inoculation of the seeder birds might have failed and an oral uptake of droppings from colonized chickens led to a colonization, this does not reduce the importance for the practice. Rather, it reflects the circumstances affecting a colonization under field conditions. Both, the colonization of the sentinel birds as well as the seeder birds is dependent upon the initial colonization of the seeder birds via inoculation. The dependency between seeder birds and sentinel birds and the resulting colonization with ESBL- *E. coli* was described by Ceccarelli et al. (2017) and is neglected in our colonization model. Moreover, besides our housing conditions, the sampling frequency does not allow to draw a conclusion of the transmission rate of ESBL- and AmpC- producing Enterobacteriaceae: our study is focused on the outcome of a practical orientated colonization.

Since the genes encoding for ESBLs and AmpCs are frequently located on plasmids (Carattoli, 2013), the possibility of a horizontal gene transfer (HGT) to other Enterobacteriaceae has to be considered. Even in the absence of antimicrobial selection pressure a transmission of resistance genes is possible (Smet et al., 2011). To ensure a reliable detection of our *E. coli* strains, a chromogenic agar was used and colony morphologies were checked up on their belonging to both strains. Additionally, in all digestive tract samples taken at necropsy, an agar plate without antibiotics was used to quantify the total count of *E. coli* colonies (data not shown). The total count of *E. coli* was congruent with the sum of our quantified ESBL- and AmpC- strains in all trails. Furthermore, out of the cecal content, colonies from the selective agar plates for the growth of the ESBL- and AmpC- *E. coli* strains were randomly selected and checked for their phenotypic antimicrobial resistances using VITEK 2. The profile of the examined colonies corresponded to those of our inoculation strains. The possibility of a HGT to other *E. coli* strains resident in the digestive tract cannot be entirely ruled out, but a colonization based on *in vivo* HGT seems to be unlikely, as in all colonization dosage trials equivalence of the cecum samples was shown. An equal HGT to other *E. coli* strains harboring the same resistance profile in all trials is highly unlikely, taking into consideration the chromosomally encoded resistances of our ESBL- *E. coli* strain.

Our results confirm the hypothesis that the uptake of only a few bacteria is sufficient to colonize broiler chickens with ESBL- and AmpC- producing Enterobacteriaceae. In fact, an inoculation of 10^1^ cfu *E. coli* leads to a colonization of broiler chickens. Due to a continuous cleaning and disinfection regime, the bacterial load at every level of the broiler production chain is supposed to be minimized. Nevertheless, there is still remaining bacteria detectable after cleaning and disinfection (Luyckx et al., 2015). Furthermore, ESBL- and AmpC- producing bacteria are found frequently (Dierikx C. M. et al., 2013; Daehre et al., 2018a). Even if there is no detailed information available about the detected quantities of these resistant bacteria after cleaning and disinfection, it is practically impossible to achieve procedures that will result in such a very low bacterial load, that a colonization of broiler chickens from the subsequent flock does not take place. In addition, the process of colonization with resistant bacteria can be greatly strengthened by applying selection pressure through antimicrobial treatment of the birds during the fattening period. The importance of cleaning and disinfection measures in broiler fattening stables is underlined by Schulz et al. (2016). The study by Schulz et al. points out, that antimicrobial resistant *E. coli* is able to survive for decades in dust samples with concentrations up to 10^4^ cfu per gram dust.

In our study, an instillation into the crop with 10^1^ cfu *E. coli* led to an equivalent colonization of broiler chickens as the higher tested doses. These data were obtained by inoculating every single animal of the group. To obtain data closer to real farming conditions, we conducted a seeder-bird model by inoculating only one fifth of the animals (seeder birds) in a group with our defined minimal colonization dosage of 10^2^ cfu *E. coli*. In literature, different methods of administration of *E. coli* to poultry are described. Besides an oral inoculation into the beak (Wang et al., 2017), a spray application over the nose and eyes of the birds and a treatment of the feeders has been implemented (Huff et al., 2011). A safer method to ensure the uptake of the bacterial suspension is an instillation into the crop. Thereby, to mimic the route of natural bacterial infection in animal trials, the seeder-bird model is frequently used and described for different enteral bacteria, such as *E. coli, Salmonella enterica* and *Campylobacter* spec. (Ratert et al., 2015; Schneitz and Hakkinen, 2016; Ceccarelli et al., 2017). The non- inoculated, susceptible sentinel birds mirror the animals naturally infected by the oral uptake of resistant bacteria from their surrounding environment after they were shed by the seeder birds. In the course of this, a variety of different ratios is known and a ratio of 1:5 seeder- to sentinel birds is more often described for the inoculation with Enterobacteriaceae (Methner et al., 2011; de Cort et al., 2015; Kilroy et al., 2015). Using this relation led to a complete colonization of all birds within our group. For us, with regard to future studies, a colonization in combination with an approximation to real farming conditions was the most important. As there is no data available about the number of initially colonized broiler chickens with resistant bacteria through contaminated farm environment, other ratios were not tested. Lower ratios of inoculated to non-inoculated broiler chickens might lead to lower prevalences of the flocks but can still constitute a source for contamination in further steps of the broiler production chain. In this connection, an introduction of resistant bacteria and cross- contamination at slaughterhouse level was proven by von Tippelskirch et al. (2018). As another consequence, remaining bacteria in the fattening stable after cleaning and disinfection is a source for the colonization of the subsequent broiler flock as described above.

After 35 days of the seeder-bird trial, the bacterial count observed in all necropsy samples taken is similar for all broilers, irrespective of being an artificially inoculated seeder bird or naturally colonized sentinel bird. This clearly shows the impact of the oral (re-) colonization with ESBL- and AmpC- producing Enterobacteriaceae during the ongoing trial. Even if the inoculation of only one fifth of the broilers initially resulted in a higher percentage of colonized seeder birds, observed in the cloacal swabs taken, the oral uptake of resistant bacteria from their surrounding environment seems to be of great importance for the colonization. In this way, all sentinel birds achieved a comparable colonization as the seeder birds over a period of 35 days. Interestingly, the bacterial count in all necropsy samples from the seeder-bird group is significantly lower compared to the dosage trial using 10^2^ cfu *E. coli*. A first possible explanation is the different number of ESBL- and AmpC- inoculated broilers per trial. The oral colonization of only one fifth of the broilers in the seeder-bird model initially leads to less shedding of resistant bacteria into the animal housing. Thus, the contamination of the environment with the bacterial strains and in consequence, the oral (re-) colonization of the birds in the seeder-bird group is lower compared to the dosage trial using 10^2^ cfu *E. coli*. The lower bacterial contamination of the environment might also explain the lower initial prevalence observed in the colonization dosage trials using 10^1^ and 10^2^ cfu ESBL- and AmpC- *E. coli* compared to 10^3^ and 10^4^ cfu. However, a reasonable explanation for the different colonization levels in the seeder-bird trial compared to the trial using 10^2^ cfu *E. coli* are the durations of the trials. With regard to strict animal welfare norms, a duration of 2 weeks was found to be adequate to demonstrate a colonization of our chickens with the *E. coli* strains in the colonization dosage trials. After defining the minimal colonization dosage of 10^2^ cfu *E. coli*, a duration of 5 weeks was chosen for the subsequent seeder-bird trial, to mimic real farming conditions. This discrepancy between the experimental periods leads to a change in microbial flora of the broilers gastrointestinal tract. Up to an age of 49 days significant differences in the composition of the cecal content for Ross-hybrids were shown by Lu et al. (2003). This is in agreement with other studies postulating a change of the predominant bacteria in broilers at older age (Amit-Romach et al., 2004; Crhanova et al., 2011). These changes in the microbial flora might lead to a competition of ESBL- and AmpC- producers with other bacteria, resulting in a decrease of ESBL- and AmpC- colonization.

As the reservoir for ESBL- and AmpC- producing *E. coli*, the cecum has a predominant role to evaluate the colonization of the broilers in the final necropsy. In contrast to the other samples of the colonization dosage trials taken during necropsy, the values gained from the content of the cecum are comparable between the trials. Whereas a fermentation of the bacteria takes places in both ceca, the content of crop and jejunum do simply reflect the recent uptake of the resistant bacteria out of the surrounding. This is strongly influenced by the feeding and drinking behavior of the broilers immediately before the necropsy. Because of the contamination with feces, an increased pecking in the litter leads to a large intake of bacteria compared to a pecking out of the feeding troughs or drinking and provides a reasonable explanation for the different bacterial counts observed in crop and jejunum samples.

Without applying any antimicrobial selection pressure to the birds, a colonization was achieved in our trials using a low dose of ESBL- and AmpC- producing *E. coli*. The usage of antibiotics in animal farming is discussed controversially, with studies indicating an impact of antibiotic treatment on co-selection and occurrence of resistant bacteria (Costa et al., 2009; Persoons et al., 2011; Dierikx C. M. et al., 2013). In recent years, an increasing number of studies are concluding that the occurrence of ESBL- and AmpC- producing bacteria in fattening chickens is not related to antibiotic treatment (Hiroi et al., 2012; Huijbers et al., 2016; Projahn et al., 2018). Our study reinforces these findings, showing a colonization even of the susceptible sentinel birds using a low dose of ESBL- and AmpC- producing *E. coli* without applying any antimicrobial selection pressure. In support of this, numerous studies are reporting a carriage of ESBL- and AmpC- resistant bacteria in wild bird species that have never been faced with any antibiotics before (Guenther et al., 2012; de Cort et al., 2015; Alcalá et al., 2016). Both of our inoculation strains were derived from a previous research project on ESBL- and AmpC- *E. coli* from chicken farming in Germany, thus presenting actual strains occurring in broiler chicken production (Falgenhauer et al., 2016; Hering et al., 2016). Besides that, ST10 and ST410 were chosen due to their ubiquitous nature as ST10 presents an ancient sequence type often present in livestock farming. ST410 was chosen due to its recent emergence as high risk clone in Germany and worldwide (Falgenhauer et al., 2016; Schaufler et al., 2016, 2019).

As a limitation, we investigated only one animal group per colonization dosage and we have no information on the variation between groups with the same colonization dosage. Although the inoculation of four different colonization dosages verified the general method, a repetition of the equal dosage or inoculation ratio of the broiler chickens might result in higher or lower initial colonization rates. Furthermore, an intra-herd correlation between the broiler chickens of one group is present and the results of the birds within one group are dependent upon each other. The effects shown can be group-specific and through a possible underestimation of variance in our analysis the results cannot be generalized completely.

Nonetheless, we showed a colonization of broiler chickens with a low colonization dosage of 10^2^ cfu *E. coli* and a small number of orally inoculated broilers in the seeder-bird model. This might be a feasible explanation for the global spread of ESBL- and AmpC- producing enterobacteria in conventional broiler farms. With regard to an assumed transmission of resistant bacteria to humans through close contact to colonized broilers or the consumption of contaminated retail meat, further steps for the reduction of ESBL- and AmpC- producing bacteria have to be considered. Consequently, based on this practical orientated colonization model, different hygiene- and management measures as well as gut microbiota influencing measures are currently being investigated as potential intervention strategies to reduce the colonization of broilers.

## Data Availability

All datasets generated for this study are included in the manuscript/Supplementary Files.

## Author Contributions

SG, UR, AB, and AF designed the study. CR, SG, and AB performed the samplings and laboratory work. RM and CR performed the statistical analysis. CR evaluated the final data and wrote the manuscript. All authors have read and approved the final draft of the article.

### Conflict of Interest Statement

The authors declare that the research was conducted in the absence of any commercial or financial relationships that could be construed as a potential conflict of interest.

## Supplementary Material

Figure S1 Colonization of broiler chickens during the four colonization dosage trials 10^1^–10^4^ cfu/*E. coli* **(A–D)** and the seeder birds and sentinel birds of the seeder-bird model trial **(E,F)** using a semiquantitative measurement with categories from 0 to 7: 0 = no growth; 1 ≤ 10 cfu *E. coli*; 2 = between 1 and 3; 3 ≤ 100 cfu *E. coli*; 4 = between 3 and 5; 5 > 100 cfu *E. coli*; 6 = between 5 and 7; 7 = agar plate overgrown; d p.i. = days post inoculation.

Table S1 Mean values and confidence intervals of digestive tract samples from colonization dosage groups (10^1^–10^4^ cfu/*E. coli*) and seeder-bird model attained at necropsy.
