# Supplementary figures and images for: Low Dose Colonization of Broiler Chickens With ESBL-/AmpC- Producing Escherichia coli in a Seeder-Bird Model Independent of Antimicrobial Selection Pressure
Source: Front Microbiol. 2019 Sep 13;10:2124. doi: 10.3389/fmicb.2019.02124 (PMC6753873; doi:10.3389/fmicb.2019.02124)

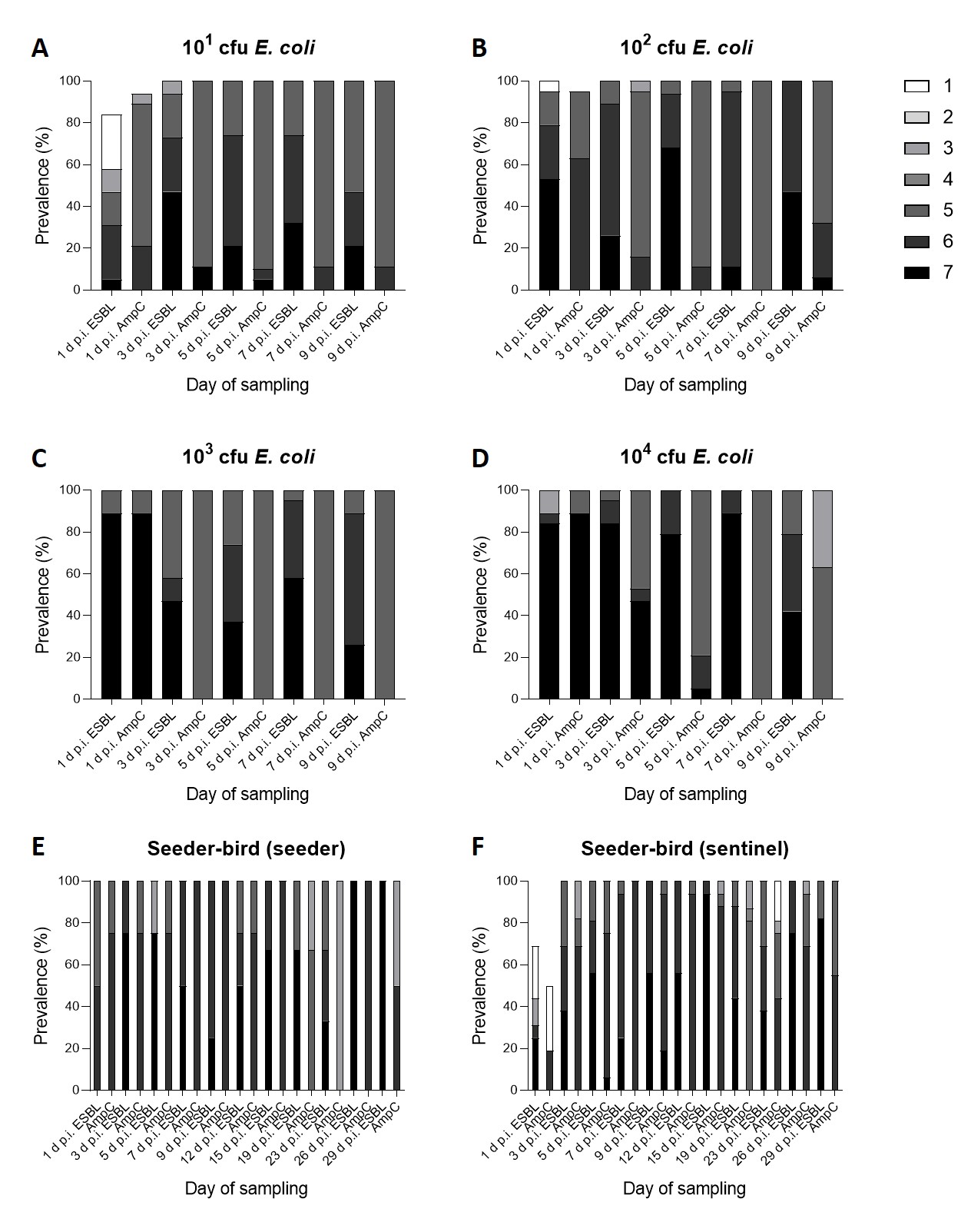

Supplement: Figure S1 — Colonization of broiler chickens during the four colonization dosage trials 101–104 cfu/E. coli (A–D) and the seeder birds and sentinel birds of the seeder-bird model trial (E,F) using a semiquantitative measurement with categories from 0 to 7: 0 = no growth; 1 ≤ 10 cfu E. coli; 2 = between 1 and 3; 3 ≤ 100 cfu E. coli; 4 = between 3 and 5; 5 > 100 cfu E. coli; 6 = between 5 and 7; 7 = agar plate overgrown; d p.i. = days post inoculation. [file Image_1.JPEG]
