# Supplementary material for: Low Dose Colonization of Broiler Chickens With ESBL-/AmpC- Producing Escherichia coli in a Seeder-Bird Model Independent of Antimicrobial Selection Pressure
Source: Front Microbiol. 2019 Sep 13;10:2124. doi: 10.3389/fmicb.2019.02124 (PMC6753873; doi:10.3389/fmicb.2019.02124)
Supplement: Table S1 — Mean values and confidence intervals of digestive tract samples from colonization dosage groups (101–104 cfu/E. coli) and seeder-bird model attained at necropsy. [file Table_1.DOCX]

Supplementary Material

# Supplementary Table

Table S1: Mean values and confidence intervals of digestive tract samples from colonization dosage groups (10^1^ - 10^4^ cfu/*E. coli*) and seeder-bird model attained at necropsy.

| **Sample** |  | **Crop** | | | |  | **Jejunum** | | | |  | **Cecum** | | | |  | **Colon** | | | |
| --- | --- | --- | --- | --- | --- | --- | --- | --- | --- | --- | --- | --- | --- | --- | --- | --- | --- | --- | --- | --- |
| Strain |  | 10716 | | 10717 | |  | 10716 | | 10717 | |  | 10716 | | 10717 | |  | 10716 | | 10717 | |
|  |  | Mean | ± CI | Mean | ± CI |  | Mean | ± CI | Mean | ± CI |  | Mean | ± CI | Mean | ± CI |  | Mean | ± CI | Mean | ± CI |
| 10^1^ cfu/*E. coli* |  | 6.77 | 7.07  6.48 | 6.68 | 6.96  6.39 |  | 4.16 | 4.80  3.51 | 4.39 | 4.91  3.86 |  | 8.94 | 9.27  8.61 | 8.62 | 8.94  8.29 |  | 7.05 | 7.43  6.67 | 7.07 | 7.39  6.76 |
| 10^2^ cfu/*E. coli* |  | 5.93 | 6.53  5.33 | 5.40 | 6.11  4.68 |  | 5.97 | 6.57  5.36 | 5.84 | 6.44  5.25 |  | 9.22 | 9.31  9.14 | 8.64 | 8.73  8.54 |  | 7.91 | 8.29  7.54 | 7.06 | 7.40  6.72 |
| 10^3^ cfu/*E. coli* |  | 9.08 | 9.87  8.30 | 8.59 | 9.37  7.82 |  | 8.18 | 9.42  6.95 | 8.33 | 9.52  7.15 |  | 8.84 | 8.90  8.74 | 8.69 | 8.83  8.54 |  | 7.28 | 7.59  6.96 | 7.15 | 7.52  6.78 |
| 10^4^ cfu/*E. coli* |  | 4.96 | 5.18  4.73 | 3.93 | 4.25  3.60 |  | 3.99 | 1.43  2.54 | 3.68 | 1.62  2.69 |  | 9.07 | 9.14  9.00 | 8.32 | 8.47  8.17 |  | 7.15 | 7.55  6.75 | 6.67 | 7.07  6.27 |
|  |  |  |  |  |  |  |  |  |  |  |  |  |  |  |  |  |  |  |  |  |
| *Seeder-bird model* |  | 4.39 | 4.73  4.06 | 4.70 | 4.98  4.41 |  | 3.05 | 3.76  2.31 | 3.89 | 4.46  3.32 |  | 6.69 | 7.10  6.29 | 6.57 | 6.96  6.17 |  | 5.29 | 5.66  4.98 | 5.32 | 5.67  4.98 |
| Seeder birds |  | 4.35 | 5.00  3.70 | 4.67 | 5.33  4.00 |  | 2.23 | 4.25  1.20 | 2.99 | 5.40  1.57 |  | 6.76 | 6.99  6.53 | 7.06 | 8.04  6.09 |  | 4.66 | 5.48  3.83 | 4.86 | 5.68  4.05 |
| Sentinel birds |  | 4.40 | 4.78  4.02 | 4.70 | 5.02  4.39 |  | 3.21 | 3.98  2.43 | 4.06 | 4.52  3.60 |  | 6.68 | 7.16  6.19 | 6.47 | 6.89  6.05 |  | 5.41 | 5.79  5.03 | 5.41 | 5.77  5.04 |

10716 = ESBL- *E. coli*, 10717 = AmpC- *E. coli*; all data shown are log10 transformed (log10 cfu/g); ± CI = ± 95% CI
